# Intestinal transcriptional profiling reveals fava bean-induced immune response in DBA/1 mice

**DOI:** 10.1186/s40659-019-0216-9

**Published:** 2019-03-01

**Authors:** Guankui Du, Man Xiao, Qiwei Zhu, Chen Zhou, Ao Wang, Wangwei Cai

**Affiliations:** 1 Department of Biochemistry and Molecular Biology, Hainan Medical College, Haikou, 571199 China; 2 Biotechnology Major, Hainan Medical College, Haikou, 571199 China

**Keywords:** Fava beans, Differentially expressed genes, DEGs, Immune response, Total IgE, Histamine

## Abstract

**Background:**

Fava beans (FBs) have long been used as food, and their principal disadvantage is derived from their haemotoxicity. We hypothesized that FB ingestion alters the intestinal gene expression pattern, thereby inducing an immune response.

**Results:**

In-depth sequence analysis identified 769 differentially expressed genes (DEGs) associated with the intestine in FB-treated DBA/1 mouse intestines. The identified genes were shown to be associated with biological processes (such as response to stimulus and immune system processes), human disease pathways (such as infectious diseases, endocrine and metabolic diseases, and immune diseases), and organismal system pathways (such as the digestive system, endocrine system, environmental adaptation, and immune system). Moreover, plasma total immunoglobulin E (IgE), histamine, interleukin (IL)-4 and IL-13 levels were significantly increased when the mice were treated with FBs.

**Conclusions:**

These results demonstrated that FBs affect the intestinal immune response and IgE and cytokine secretion in DBA/1 mice.

**Electronic supplementary material:**

The online version of this article (10.1186/s40659-019-0216-9) contains supplementary material, which is available to authorized users.

## Background

Fava bean (FB) have long been used as food and feed because of their rich source of proteins and energy [1, 2]. Therefore, FB is prevalent in many areas, such as the Middle East and South America [1]. However, the main disadvantage of FB is derived from its haemotoxicity, which can induce favism in individuals with glucose 6-phosphate dehydrogenase (G6PD) deficiency [3].

Favism is an acute haemolytic disease that occurs 24–48 h after FB ingestion [4]. Favism occurs more frequently in 1- to 5-year-old children, and it is rare in adults. Several hypotheses have been proposed for the onset of favism, such as oxidized substances in FBs (such as divicine and isouramil), hereditary factors, and immunological factors [5–7]. Although all patients with favism are G6PD-deficient, many patients with G6PD deficiency can ingest FBs without clinical symptoms [4]. Therefore, factors other than G6PD deficiency require further study.

Food allergies are a severe health problem in approximately 6% of infants younger than 3 years [8]. Immunoglobulin E (IgE) mediates acute allergic reactions, such as urticaria, vomiting, wheezing and anaphylaxis, that manifest after the ingestion of food [9, 10]. After allergic sensitization has occurred, CD4^+^ TH2 cells secrete anti-inflammatory molecules [interleukin (IL)-4 and IL-13], which result in the overproduction of IgE [11, 12]. IgE binds the high-affinity IgE receptor FcεRI on the surface of mast cells [13, 14]. Subsequently, the mast cells rapidly release proinflammatory mediators (such as histamine), which induce the acute phase of the allergic response [15].

Notably, a few case reports have shown allergic reactions to FB [16]. Damiani et al. [17] showed occupational allergies induced by volatile substances released from FB. Moreover, Bousfiha and Aarab [18] showed that a high proportion of the Moroccan population has positive values for the IgE specific to the FB protein. Recently, Kumar et al. showed that mice have a severe allergic response to FB, and undigested FB proteins may be the allergen related to allergic responses in mice and humans. It has been reported that the mucosal immune system in the intestine plays an important role in food allergies [19, 20]. Therefore, we hypothesized that FB affects the expression of genes involved in the immune response in the intestinal tract to trigger allergic reactions. This study aimed to determine the gene expression profile of the intestine in DBA/1 mice after FB ingestion.

## Methods

### Animals and treatment

Animals were treated as approved by the Hainan Medical College Animal Research Committee. A total of 16 3- to 4-week-old male DBA/1 mice were obtained from the Model Animal Research Center of Nanjing University (Nanjing, China). All mice were housed in a room with a controlled environment (a temperature of 22 ± 1 °C, relative humidity of 50 ± 5%, and a 12-h light/12-h dark cycle). Food and water were provided ad libitum. The mice were randomized into a control group and an FB-treated group (n = 8 mice per group). As conducted in our previous study, the FB-treated mice underwent intragastric administration of 10 g/kg body weight cooked FB homogenate (20% w/v), which could significantly induce oxidative stress [21], and the control mice underwent intragastric administration of the same volume of water. After 8 h, blood samples were obtained from the ocular vein for analysis of the content of IgE, histamine, IL-1, and IL-13. The mice were sacrificed under chloral hydrate anaesthesia. Approximately 100 mg intestinal samples, obtained 1 cm below the stomach, were sliced and frozen for deep sequencing analysis and reverse transcription PCR (RT-PCR).

### Biochemical assay

The contents of plasma IgE, histamine, IL-1, and IL-13 were determined by ELISA assays using commercial kits (Elabscience Biotechnology Co., Ltd., Wuhan, China; IgE (E-EL-M0691c), histamine (E-EL-0032c), IL-4 (E-EL-M0043c), and IL-13 (E-EL-M0727c)) according to the manufacturer’s instructions.

### RNA isolation

Total RNA was isolated by a TRIzol kit (Invitrogen, NY, USA) according to the manufacturer’s instructions. Then, the total RNA samples from each group were further extracted in triplicate by an Oligotex mRNA Mini Kit (Qiagen, Valencia, CA) for RNA-Seq.

### RNA-Seq

A BGISEQ-500 system (BGI, Shenzhen, China) was used for library construction and sequencing. The reads were matched with the reference genome of *M. musculus* (GRCm38 version 74.38; ensemble.org/Mus_musculus) by hierarchical indexing for spliced alignment of transcripts (HISAT) [22]. RESM software was used for gene expression analysis [23]. The differential expression of genes was isolated by a significance threshold (absolute value of a log2 ratio ≥ 1 and an FDR < 0.001).

### Gene ontology (GO) and pathway enrichment analysis

DAVID software (http://david.abcc.ncifcrf.gov/) was used for gene ontology (GO) and kyoto encyclopedia of genes and genomes (KEGG) analysis [24, 25]. The enriched pathway was selected by the hypergeometric test with the Bonferroni correction.

### Analysis of mRNA expression by RT-PCR

The sequence data were validated by RT-PCR. A total of 2 μg of RNA was used to synthetize the first strand by a reverse transcription system (Promega, Wisconsin, USA) according to the manufacturer’s protocol. The PCR reactions (20 μL) contained 20 ng of template cDNA, 10 μL 2× Master Mix (Sigma-Aldrich, Dorset, England), and 10 μΜ of each forward and reverse primer (Additional file 1: Table S1) (Sangon, Shanghai, China).

### Statistical analysis

All data are presented as the mean ± standard error (SE). Significant differences among the groups were analysed using Student’s t-test. A P-value < 0.05 was considered statistically significant. All analyses were performed using GraphPad Prism version 5.0 (GraphPad Software, San Diego, CA).

## Results

### Differentially expressed intestinal genes after FB ingestion

**Fig. 1:**
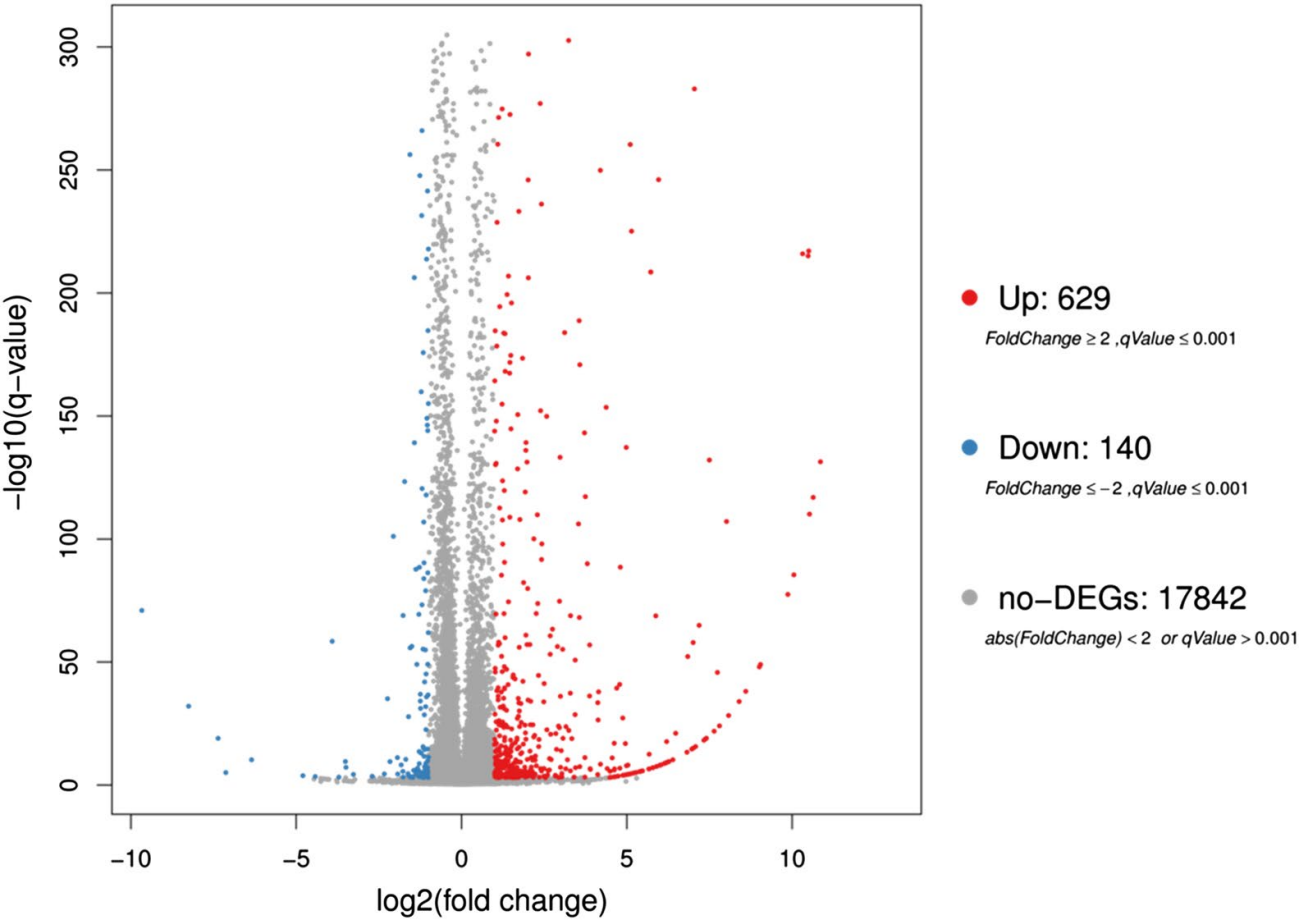
Volcano plot of gene expression differences between the normal and FB-treated mouse groups. The X-axis represents the log2 transformed fold change. The Y-axis represents the − log10 transformed significance. The red points represent upregulated DEGs. The blue points represent downregulated DEGs. They grey points represent non-DEGs

Based on strict statistical criteria (median FDR < 0.01; fold change > 2), SAM analysis identified 769 differentially expressed genes (DEGs) of the intestine between the control and FB-treated groups (Fig. 1). Of these genes, 629 were significantly upregulated, and 140 were significantly downregulated (Additional file 2: Table S2).

### Gene ontology (GO) analysis of DEGs

**Fig. 2:**
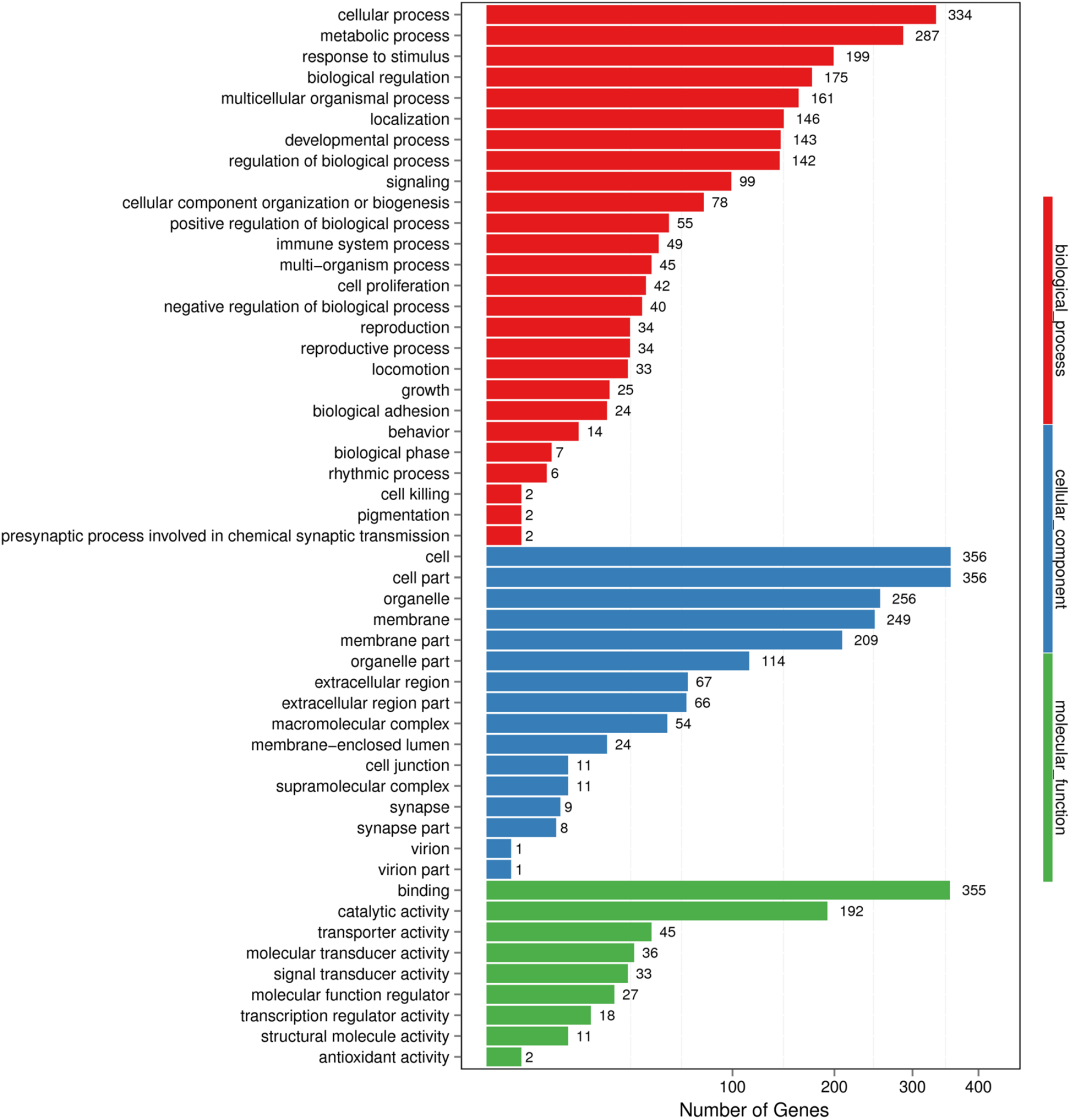
GO classification of DEGs. The X-axis represents the number of DEGs. The Y-axis represents the GO term

To obtain insights into the functions of the identified DEGs, gene ontology (GO) analysis was performed (Fig. 2). These genes were shown to be mainly involved in the following biological process categories: response to the stimulus, biological regulation, multicellular organismal process, localization, developmental process, regulation of biological process and immune system process. These genes were shown to be mainly involved in the following molecular function categories: binding, catalytic activity, transporter activity, molecular transducer activity, signal transducer activity, molecular function regulator, transcription regulator activity, structural molecule activity, and antioxidant activity. Additional file 3: Table S3 lists the differentially expressed genes implicated in each of the functions mentioned above.

### Pathway analysis of differentially expressed genes

**Fig. 3:**
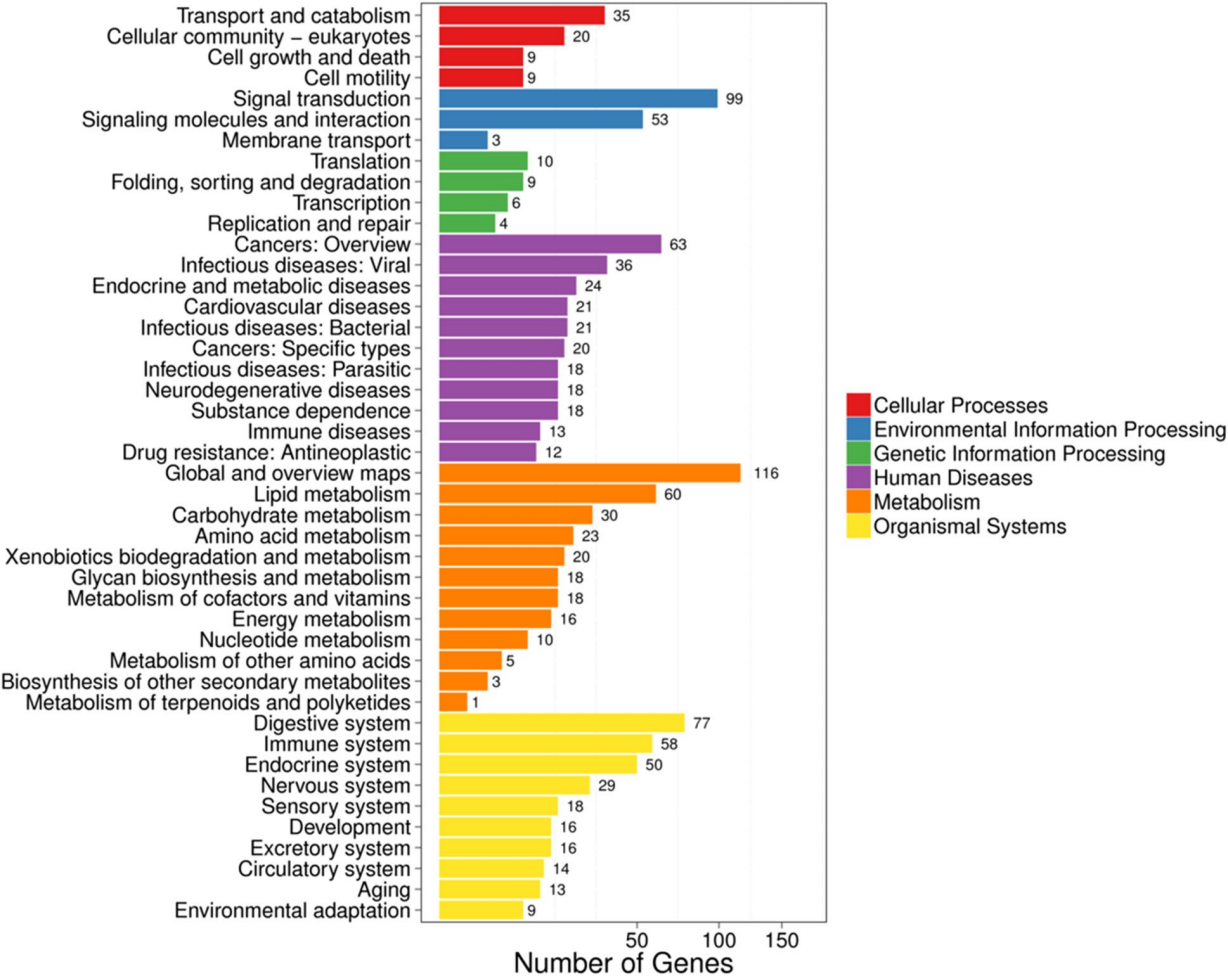
Pathway classification of DEGs. The X-axis represents the number of DEGs. The Y-axis represents the functional classification of KEGG. There are seven branches for KEGG pathways, including cellular processes, environmental information processing, genetic information processing, human disease (for animals only), metabolism, organismal systems and drug development

We performed KEGG pathway classification and functional enrichment of the DEGs. As shown in Fig. 3 and Additional file 4: Table S4, the DEGs were associated with 6 branches for KEGG pathways: cellular processes, environmental information processing, genetic information processing, human disease (for animals only), metabolism, organismal systems and drug development. Notably, DEGs associated with human disease-related categories are as follows: infectious diseases: viral, endocrine and metabolic diseases, cardiovascular diseases, infectious diseases: bacterial, infectious diseases: parasitic, neurodegenerative diseases, and immune diseases. Furthermore, DEGs associated with organismal system-related categories are as follows: digestive system, ageing, circulatory system, development, endocrine system, environmental adaptation, excretory system, immune system, nervous system, and sensory system.

### RT-PCR verification of the sequencing results

**Fig. 4:**
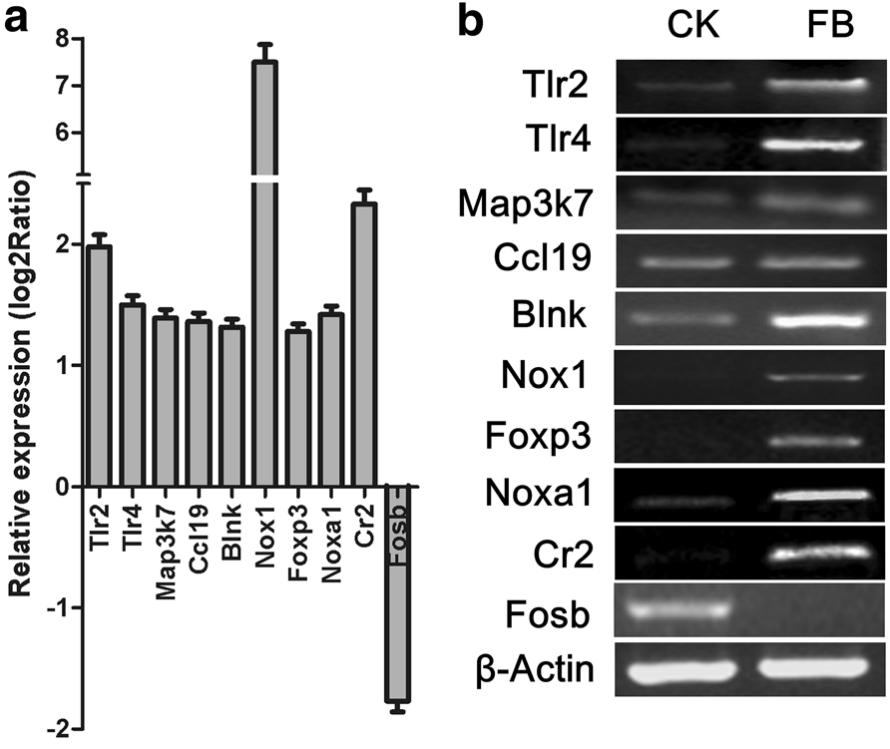
Confirmation of the microarray results by RT-PCR. Gene expression of Tlr-2, Tlr-4, Map3k7, Ccl19, Blnk, Nox1, Foxp3, Noxa1, Cr2 and Fosb in mice on a control diet and mice supplemented with FBs was analysed by microarray (**a**) and RT-PCR (**b**) using the stable reference gene β-Actin. The data are presented as the mean ± SE (n = 6)

For validation of the sequencing data, a group of genes known to be directly or indirectly implicated in the immune response was used for RT-PCR. These genes included Tlr2, Tlr4, Map3k7, Foxp3, Nox1, Noxa1, Cr2, Ccl19, Blnk and Fosb (Fig. 4). Tlr2, Tlr4, Map3k7, Foxp3, Nox1, Noxa1, Cr2, Ccl19, and Blnk were observed to be upregulated in the intestines of DAB/1 mice after FB ingestion, while Fosb was downregulated. There was excellent agreement between the sequencing and RT-PCR data.

### Gene expression showed an increase in immune responses after FB ingestion

Functional pathway enrichment identified 26 immune response pathways, which included 64 genes (Table 1). The main immune response pathways include inflammatory bowel disease (IBD), leukocyte transendothelial migration, complement and coagulation cascades, the Fc epsilon Ri signalling pathway, the chemokine signalling pathway, the toll-like receptor signalling pathway, the B cell receptor signalling pathway, systemic lupus erythematosus, platelet activation, and the IL-17 signalling pathway.

**Table 1:** The genes involve in immune responses pathways after FB ingestion

Immune response related pathway	GeneID
Inflammatory bowel disease (IBD)	24088, 53791, 21898, 20371
Leukocyte transendothelial migration	13057, 226654, 237038, 57257, 241275, 16409, 14677, 18710, 58187, 216033, 50490, 12737
Complement and coagulation cascades	69864, 78354, 11537, 16409, 12902, 14058, 74145, 14067, 232345
Fc epsilon RI signaling pathway	57257, 18710, 211429, 271844, 102238433
Chemokine signaling pathway	18829, 57257, 100504362, 24047, 14677, 11514, 18710, 245386, 100862177, 65956
Toll-like receptor signaling pathway	24088, 18710, 53791, 21897, 21898, 224419
Toll and Imd signaling pathway	407243, 245381, 224419
B cell receptor signaling pathway	17060, 57257, 18710, 12902
Systemic lupus erythematosus	15077, 319164, 319191, 319167, 319150, 319184
Platelet activation	14677, 11514, 18710, 211429, 271844, 102238433, 27220
IL-17 signaling pathway	14282, 70031, 57890, 224419
Graft-versus-host disease	110557, 15051
NOD-like receptor signaling pathway	13057, 237038, 78688, 21898, 100417831, 381308, 12374, 50490, 224419
Allograft rejection	110557, 15051
Fc gamma R-mediated phagocytosis	111002, 57257, 18710
RIG-I-like receptor signaling pathway	67664, 224419
Natural killer cell mediated cytotoxicity	57257, 18710, 379043, 19368
Cytosolic DNA-sensing pathway	232934, 381308
T cell receptor signaling pathway	57257, 18710, 224419
Rheumatoid arthritis	24088, 21898
Antigen processing and presentation	73442, 110557, 15051
Hematopoietic cell lineage	16409, 12902
Autoimmune thyroid disease	110557, 15051
Th1 and Th2 cell differentiation	67701, 19668
Primary immunodeficiency	17060
Th17 cell differentiation	381538, 20371

### IgE and cytokine levels increased after FB ingestion

**Fig. 5:**
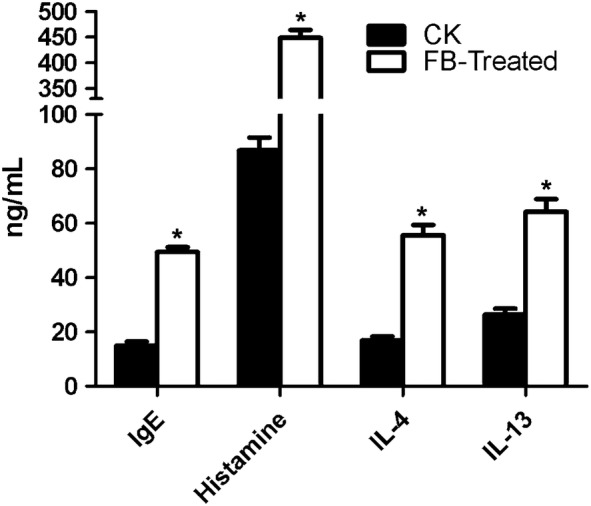
The levels of IgE and cytokines in mouse blood were analysed after treatment with fava beans. The data are presented as the mean ± SE (n = 8). Asterisk indicates a significant difference (*P *< 0.05) between the control and FB-treated groups

Since most of the genes induced in mice treated with FB were shown to be involved in immune and inflammatory responses, we investigated plasma total IgE, histamine, IL-4 and IL-13 (Fig. 5). Marked enhancement in the levels of IgE (3.3-fold, *P *< 0.05) and histamine (5.1-fold, *P *< 0.05) were observed in the plasma of FB-treated mice compared with the control mice. The levels of the cytokines IL-4 and IL-13 significantly increased by 3.3-fold (*P *< 0.05) and 2.4-fold (*P *< 0.05), respectively, in the plasma of FB-treated mice compared with the control mice (Fig. 5).

## Discussion

DBA/1 mice are used for the development of various murine models, such as halothane hepatitis and immune nephritis [26, 27]. Mostly, DBA/1 mice have been used as a model of autoimmunity [28]. The production of IgE has been shown to be significantly increased in collagen-induced arthritic mice [29]. Serum IgE is considered one of the essential diagnostic parameters in the diagnostic workup of food allergy [30]. DBA/1 is a suitable mouse model for immunology and inflammation research. Therefore, we employed DBA/1 mice to investigate the FB-induced immune response.

All favism patients that have ingested FBs appear to be quite ill, with pallor, jaundice, abdominal pain, dark urine and often fever. Favism can be a very severe, life-threatening form of acute haemolytic anaemia [3]. These symptoms are notably similar to an allergic reaction to food. Surprisingly, FB allergenicity was not reported until 2007 [16]. A 25-year-old Spanish female showed an allergic reaction after ingesting a sandwich containing FB flour [16]. Twelve percent of children with food allergies have an FB allergy [31]. Moreover, increased levels of total IgE and histamine have been observed in mice sensitized with FB [31], which are vital signs of systemic anaphylaxis [32]. In our present study, the levels of total IgE, histamine, IL-4, and IL-13 were significantly increased in the DBA/1 mice 8 h after FB ingestion, which is consistent with a report indicating that allergic patients exhibit symptoms within minutes to several hours after FB ingestion [33]. DBA/1 mice could serve as an alternative animal model of favism, and FBs can elicit an immune response in the DBA/1 mice.

In the present study, the BGISEQ-500 platform was used for sequencing and generated approximately 24.00 M reads per sample. A total of 19,677 genes were detected. The filter composition statistics of the raw data are shown in Additional file 5: Figure S1. Clean-read quality metrics are shown in Additional file 6: Table S5. The distribution of base quality is shown in Additional file 7: Figure S2. The samples showed good quality and provided sufficient sequencing data, indicating that most transcripts were entirely covered, and reads were evenly distributed throughout the transcript. Based on the gene expression level, 769 DEGs were identified. These genes were shown to be mainly involved in the following cellular component categories: cell, cell part, organelle, extracellular region, membrane, membrane part, membrane-enclosed lumen, organelle part, macromolecular complex, cell junction, and supramolecular complex. These genes were shown to be mainly involved in the following pathways: protein digestion and absorption, metabolic pathways, starch and sucrose metabolism, pancreatic secretion, fat digestion and absorption, arachidonic acid metabolism, carbohydrate digestion and absorption, alpha-linolenic acid metabolism, linoleic acid metabolism, metabolism of xenobiotics by cytochrome P450, pentose and glucuronate interconversions, glycerolipid metabolism, ether lipid metabolism, and nitrogen metabolism (Additional file 8: Table S6). Our previous study showed that ingestion of FBs could trigger alterations of protein, lipid and energy metabolism in mice [34]. The present study serves as a proof-of-concept that FBs could induce multiple alterations of lipid and amino acid metabolism after short-term FB ingestion.

In the present study, we identified 64 genes that were shown to be mostly associated with immune functions and processes, which agrees with our earlier observations that FBs induced differential alterations in the expression of genes related to Toll-like receptor 4 binding, the inflammatory response, the immune system process, and the acute-phase response in the mouse liver. The mRNA expression levels of the toll-like receptor (TLR) signalling pathway genes TLR1, TLR2, TLR4, TLR5, TAK1, and PI3K were significantly increased in FB-treated mice. The TLR1–TLR2 complex activates signalling events by regulating the PI3K/Akt signalling pathway and then activates transcription factors, such as nuclear factor kappa B (NF-κB) [35, 36]. TLR4 and TLR5 promote MyD88-TRAF6-TAK1 signalling to activate NF-κB or activator protein 1 (AP-1) [37, 38]. These factors bind to promoter elements of genes involved in the expression of inflammatory mediators and cytokines [39], which increase the risk of developing immune-mediated diseases, such as allergic rhinitis and autoimmune diseases [40]. A previous report showed increased expression levels of TLR2 and TLR4 in patients with inflammatory bowel diseases (IBDs), which are chronic inflammatory syndromes of the gastrointestinal tract [41]. Therefore, our results suggest that FBs might induce the TLR signalling pathway to activate the mouse inflammatory response.

Furthermore, we observed that Foxp3, CCL19, CR2, and BLNK expression levels were significantly increased in FB-treated mice. Foxp3, a crucial transcription factor for regulatory T cell function [42], participates in regulating the inflammatory immune response and could serve as a novel target candidate for the treatment of autoimmune and allergic diseases [43]. The chemokine CCL19 is critical for allergic inflammation [44, 45]. Human CR2 plays a crucial role in linking the innate and adaptive immune responses [46, 47]. B cell linker protein (BLNK) is a central linker protein involved in B cell signal transduction and might play an essential role in the variable lymphocyte receptor B cell-mediated adaptive immune response [48, 49]. Therefore, our results indicate that FBs might induce an immune response through multiple pathways.

Furthermore, we observed that Noxa1 and Nox1 expression levels were significantly increased in FB-treated mice. Noxa1 acts as a central component of NADPH oxidase (NOX), while Nox1 could affect reactive oxygen species (ROS) production, both of which might contribute to oxidative stress in allergic rhinitis [50]. Thus, the increased NOX gene mRNA expression level corroborates our previous study that FBs could induce oxidative stress in mouse plasma [34].

## Conclusions

In summary, we showed that immunoglobulin and cytokines were increased in FB-treated mice. The DEGs demonstrated that FBs induce multiple processes, such as the digestive system, endocrine system, environmental adaptation, and immune system. The TLR signalling pathway was observed to be affected by FB stress. Further research needs to be conducted to investigate the immune response in favism patients.

## Additional files

**Additional file 1: Table S1.** The PCR primer sequences.

**Additional file 2: Table S2.** SAM analysis identified 769 differentially expressed genes of the intestine between the control and FB-treated groups.

**Additional file 3: Table S3.** The differentially expressed genes implicated in each of the enriched functions.

**Additional file 4: Table S4.** KEGG pathway classification and functional enrichment of the DEGs.

**Additional file 5: Figure S1.** The filter composition statistics of raw data.

**Additional file 6: Table S5.** Clean-read quality metrics.

**Additional file 7: Figure S2.** The distribution of base quality.

**Additional file 8: Table S6.** 769 identified DEGs mainly involved in cellular component categories.

## Abbreviations

FB: fava beans
G6PD: glucose 6-phosphate dehydrogenase
IgE: immunoglobulin E
IL: interleukin
RT-PCR: reverse transcription PCR
DAVID: database for annotation, visualization and integrated discovery
DEGs: differentially expressed genes
GO: gene ontology
IBD: inflammatory bowel disease
TLR2: toll-like receptor 2
MAP3K7: mitogen-activated protein kinase kinase kinaser
FOXP3: forkhead box protein 3
NOX1: NADPH oxidase isoforms 1
NOXA1: NADPH oxidase activator 1
CR2: complement receptor type 2
CCL19: chemokines and cytokines ligands 19
BLNK: B cell linker protein
FosB: FBJ murine osteosarcoma viral oncogene homolog B
