# Supplementary material for: Intestinal transcriptional profiling reveals fava bean-induced immune response in DBA/1 mice
Source: Biol Res. 2019 Mar 1;52:9. doi: 10.1186/s40659-019-0216-9 (PMC6396536; doi:10.1186/s40659-019-0216-9)
Supplement: Supplementary file 1 — Additional file 1: Table S1. The PCR primer sequences. [file 40659_2019_216_MOESM1_ESM.docx]

|  | **Forward** | **Reverse** |
| --- | --- | --- |
| **TLR-2** | CACTGGGGGTAACATCGCTT | TGTCGGGCATCGGATGAAAA |
| **TLR-4** | AGTGGCCCTACCAAGTCTCA | GCTGCAGCTCTTCTAGACCC |
| **MAP3K7** | GAGGTGGAAGAGGTTGTCGG | TCATCCGCTCCTGGGAAGTA |
| **CCL19** | CCATCCCTGGGAACATCGTG | GTGTGGTGAACACAACAGCA |
| **BLNK** | ATAATCGATCCAGCCAGCGG | GGGAGGAACGGGAGTAGTCT |
| **NOX1** | CCTGCTCATTTTGCAACCGT | TGGAAGCAAAGGGAGTGACC |
| **FOXP3** | TGGGATCAATGTGGCCAGTC | GGCGAACATGCGAGTAAACC |
| **NOXA1** | GGACCAAGGCTGCCAATACT | GCTCGTGTGACCACACATAGAT |
| **CR2** | GGAGTGCCCATCACTTCCAA | AGGAGGAGCCTGACATCCTT |
| **FOSB** | GTGAAGACCGTGTCAGGAGG | ACGTGTAAGTAGTGCAGCCC |
| **ACTIN** | GTGAAGGTGGACAGCATGCGGTT | GAAGTGGGGTGGCTTTTAGGA |

Table S1 **The PCR primer sequences**
