# Supplementary figures and images for: Intestinal transcriptional profiling reveals fava bean-induced immune response in DBA/1 mice
Source: Biol Res. 2019 Mar 1;52:9. doi: 10.1186/s40659-019-0216-9 (PMC6396536; doi:10.1186/s40659-019-0216-9)

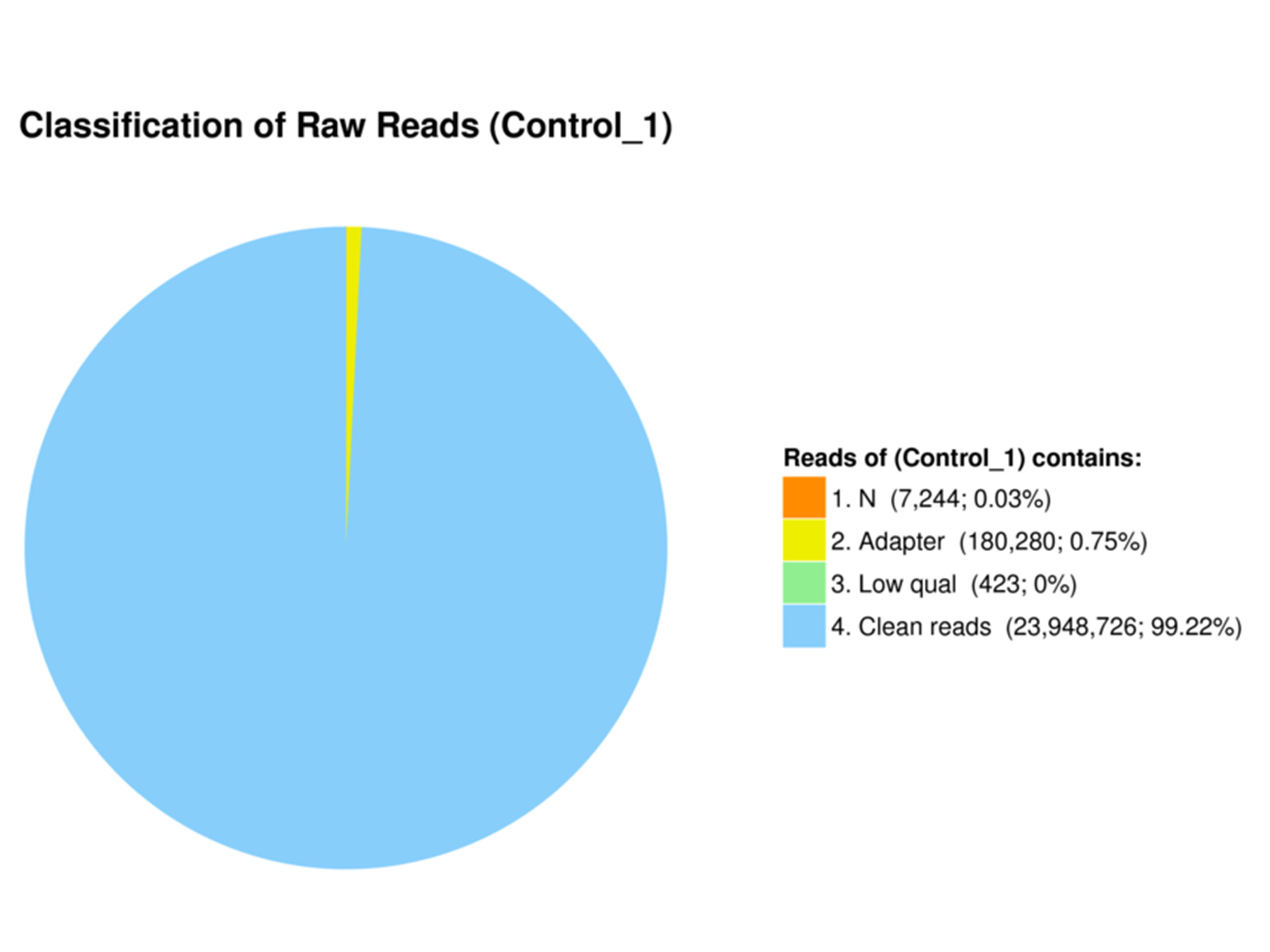

Supplement: Supplementary file 5 — Additional file 5: Figure S1. The filter composition statistics of raw data. [file 40659_2019_216_MOESM5_ESM.tif]

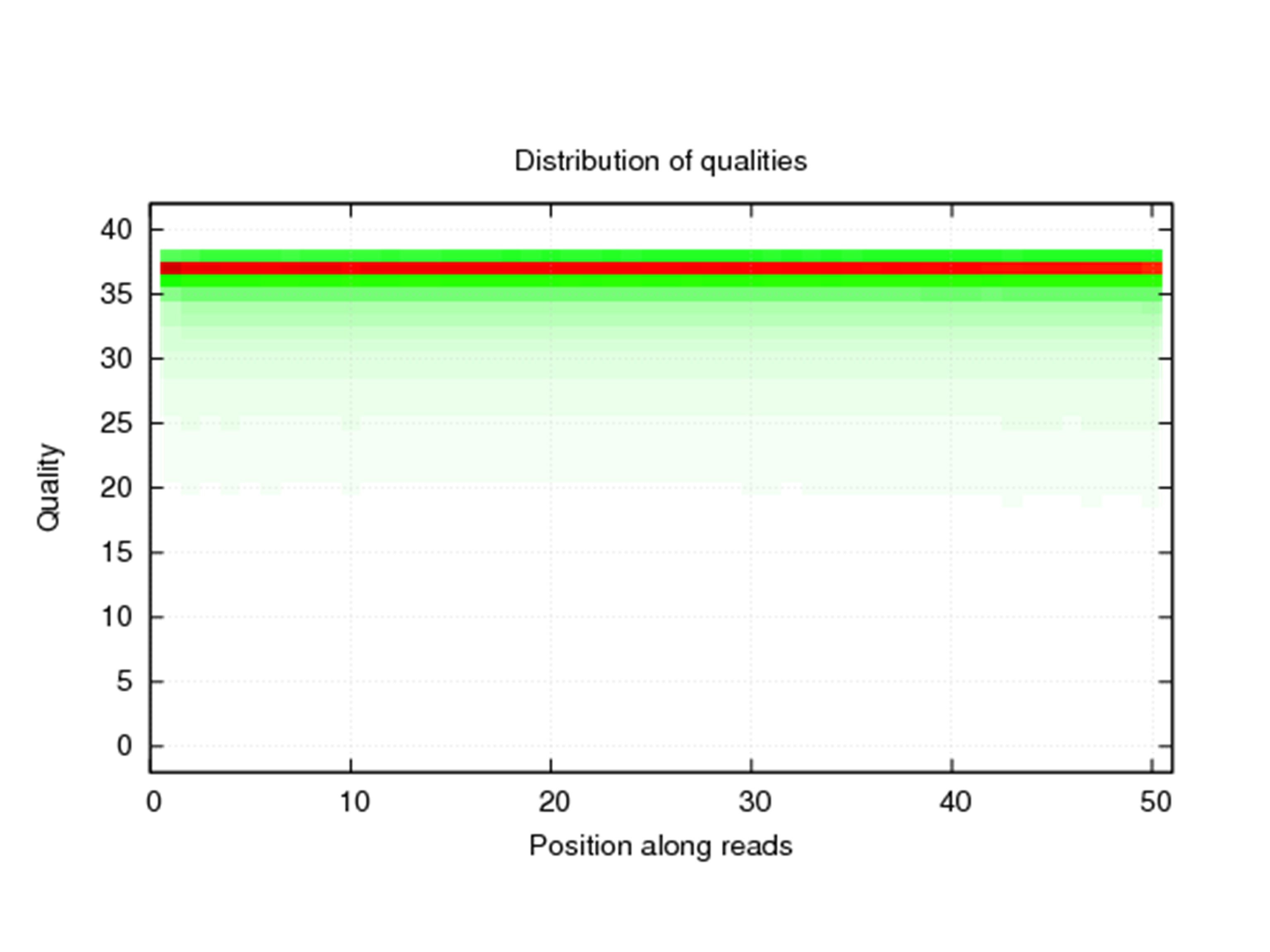

Supplement: Supplementary file 7 — Additional file 7: Figure S2. The distribution of base quality. [file 40659_2019_216_MOESM7_ESM.tif]
